# MiR-6511b-5p suppresses metastasis of pMMR colorectal cancer through methylation of CD44 by directly targeting BRG1

**DOI:** 10.1007/s12094-022-02845-4

**Published:** 2022-05-19

**Authors:** JinMing Sun, Ling Ye, Yuan Shi, XingWei Wang, XiaFei Zhao, ShengYong Ren, JunWei Fan, HuanZhang Shao, BingYu Qin

**Affiliations:** 1 Department of Critical Care Medicine, Henan Key Laboratory for Critical Care Medicine, Zhengzhou Key Laboratory for Critical Care Medicine, Henan Provincial People’s Hospital, Zhengzhou, 450003 Henan China; 2 Department of General Surgery, Shanghai General Hospital, Shanghai Jiao Tong University School of Medicine, Shanghai, China; 3 Zhengzhou University People’s Hospital, Henan University People’s Hospital, Zhengzhou, 450003 Henan China

**Keywords:** DNA mismatch repair proficient, Colorectal cancer, Metastasis, MiR-6511b-5p, Methylation

## Abstract

**Purpose:**

Distal metastases are a major cause of poor prognosis in colorectal cancer patients. Approximately 95% of metastatic colorectal cancers are defined as DNA mismatch repair proficient (pMMR). Our previous study found that miR-6511b-5p was downregulated in pMMR colorectal cancer. However, the mechanism of miR-6511b-5p in pMMR colorectal cancer metastases remain unclear.

**Methods:**

We first used quantitative real-time PCR to evaluate the role of miR-6511b-5p in colorectal cancer. Second, we conducted invasion assays and wound healing assays to investigate the role of miR-6511b-5p and CD44 in colorectal cancer cells metastases. Third, luciferase reporter assay, in situ hybridization (ISH), and immunohistochemistry assays were performed to study the relationship between miR-6511b-5p and BRG1. Finally, real-time quantitative PCR, immunohistochemistry, and chromatin immunoprecipitation (ChIP) assays were performed to analyze the relationship between BRG1 and CD44 in colorectal cancer.

**Results:**

We found that lower expression of miR-6511b-5p appeared more often in pMMR colorectal cancer patients compared with dMMR (mismatch repair deficient) cases, and was positively correlated with metastases. In vitro, overexpression of miR-6511b-5p inhibited metastasis by decreasing CD44 expression via directly targeting BRG1 in colorectal cancer. Furthermore, BRG1 knockdown decreased the expression of CD44 by promoting CD44 methylation in colorectal cancer cells.

**Conclusion:**

Our data suggest that miR-6511b-5p may act as a promising biomarker and treatment target for pMMR colorectal cancer, particularly in metastatic patients. Mechanistically, miR-6511b-5p suppresses invasion and migration of colorectal cancer cells through methylation of CD44 via directly targeting BRG1.

**Supplementary Information:**

The online version contains supplementary material available at 10.1007/s12094-022-02845-4.

## Introduction

Colorectal cancer is one of the most diagnosed cancers in the world, and approximately 50–60% of these patients develop distant metastases, mainly metastases to the liver [1, 2]. The DNA mismatch repair (MMR) system is important for the recognition and repair of DNA mismatches [3]. Human mutS homolog 2 (hMSH2), human mutL homolog 1 (hMLH1), hMSH3, hMSH6, hMLH3, and hPMS2 constitute the MMR protein family [4]. In sporadic colorectal cancer patients, about 80–90% were identified as proficient DNA mismatch repair (pMMR), microsatellite-stability (MSS), or microsatellite-instability-low (MSI-L), whereas the remaining cases account for 10–20% exhibiting microsatellite-instability-high (MSI-H), which is caused by deficient DNA mismatch repair (dMMR) [5]. In comparison with dMMR colorectal cancer cases, pMMR cases exhibit a high recurrence rate, high risk of metastasis, low survival rate, and poor prognosis [6]. In recent years, the application of immune checkpoint inhibitors has profoundly improved the medical treatment of colorectal cancer, contributing to excellent improvements in survival rates for some patients [7]. However, the majority of metastatic colorectal cancers, defined as pMMR (about 95% of metastatic colorectal cancer), fail to benefit from checkpoint inhibitors in comparison with dMMR [8]. Therefore, more attention should be paid to the molecular mechanisms of metastatic pMMR colorectal cancer. Although great efforts have been made to elucidate the underlying mechanism of colorectal cancer, the mechanisms of metastasis in pMMR colorectal cancer remain to be further explored.

MicroRNAs (miRNAs) are short non-coding RNAs capable of inhibiting the expression of protein-coding genes by partially binding to complementary sites of target mRNAs [9]. Several previous studies have reported that miRNAs are associated with many biological processes in colorectal cancer, including migration, invasion, angiogenesis, proliferation, and apoptosis [9–11]. In recent years, the relationship between MMR status and miRNA processing has also been detected [12]. For example, MLH1 contributes to miR-422a processing in HCT-116 cells and vice versa, indicating the existence of regulatory feedback between MMR status and miRNA expression [12]. Moreover, increased miR-155 was shown to correlate with dMMR colorectal cancer by inhibiting the expression of MSH2 and MLH1 [13]. However, few studies have focused on miRNAs to explore their functions in pMMR colorectal cancer. In our previous study, miR-6511b-5p was found to be downregulated in pMMR cells when compared with dMMR cells which were obtained using laser capture microdissection (LCM) from colorectal cancer tissues [14]. However, whether it plays a role in the metastasis of pMMR colorectal cancer needs to be further explored.

As a transmembrane adhesion glycoprotein, CD44 is regulated by a variety of molecules, such as transcription factors and microRNAs [15]. In addition, CD44 influences various human cancers mainly by stimulating signaling pathways that play important roles in proliferation, apoptosis, invasion, and metastasis [16]. In comparison colorectal with normal tissues, CD44 was highly expressed in primary and metastatic colorectal cancers [17]. Furthermore, CD44 was also demonstrated to enhance epithelial-mesenchymal transition (EMT), which mediates invasion and metastasis in colorectal cancer cells [17]. Nevertheless, whether miR-6511b-5p is involved in metastasis by regulating CD44 in pMMR colorectal cancer has not been explored.

In this study, we discovered that miR-6511b-5p was significantly reduced in pMMR colorectal cancer patients and correlated with metastasis. Furthermore, we examined the mechanisms of invasion and migration regulated by miR-6511b-5p and determined whether miR-6511b-5p abated the invasion and migration ability of pMMR colorectal cancer cells by inhibiting CD44. In addition, we also found that miR-6511b-5p can directly target brahma-related gene 1 (BRG1), also known as SMARCA4, a key regulator of CD44 and a major transcriptional regulator [18, 19], to suppress invasion and migration by regulating CD44 in pMMR colorectal cancer cells. We found that miR-6511b-5p might act as a diagnostic marker and contribute to the treatment of pMMR colorectal cancer through regulation of the BRG1/CD44 axis.

## Materials and methods

### Human colorectal cancer tissue collection

Colonic tissue samples from 122 patients were collected immediately after radical resection at the Department of General Surgery, Shanghai General Hospital, between 2011 and 2013. Before radical resection, no other therapies, such as chemotherapy, radiotherapy, or immunotherapy, were administered to these patients. MMR status was diagnosed using immunohistochemical analysis (IHC). The diagnosis was confirmed by two pathologists, and cancer staging was determined based on pathological findings according to the American Joint Committee on Cancer (AJCC). Informed consent was obtained from all patients, and the study was approved by the institutional review board of Shanghai General Hospital.

### Total RNA extraction and real-time quantitative PCR(RT-qPCR)

Total RNA from colorectal cancer samples and cell lines was extracted using TRIzol reagent (Invitrogen, Carlsbad, CA, USA). First-strand cDNA was synthesized using the RevertAid First Strand cDNA Synthesis Kit (Qiagen, Hilden, Germany) using 1 μg of total RNA as the template. RT-qPCR was performed using the SYBR-Green PCR Master Mix Kit (Takara, Japan) on the ViiA™ 7 system (Thermo Fisher Scientific, Waltham, MA, USA), according to the manufacturer’s instructions. The primers for miR-6511b-5p, BRG1 and CD44 were used as suggested in previous studies [17, 20, 21]. Relative mRNA expression levels were measured using the 2^−ΔΔCt^ (Ct; cycle threshold) method [22].

### Cell culture and chemicals

Four MSS human colorectal cancer cell lines, Caco-2, SW480, SW620, and HT29, were obtained from the Cell Resource Center of the Shanghai Institutes for Biological Sciences Type Culture Collection of the Chinese Academy of Sciences (Shanghai, China). The MSI-H human colorectal cancer cell line HCT116 was purchased from ATCC. All cell lines were cultured in a humidified incubator at 37 °C with 5% CO_2_ and maintained in Dulbecco’s modified Eagle medium (DMEM; Cat#C11995500BT, Gibco; Carlsbad, CA, USA) supplemented with 10% fetal bovine serum (FBS; Cat #16,000,044, Gibco; Carlsbad, CA, USA). 5-aza-2′-deoxycytidine (5-Aza-CdR) was purchased from Sigma Aldrich.

### Plasmid construction

The lentiviral vector encoding miR-6511b-5p mimics and a negative control was designed and synthesized by BioLink (Shanghai, China). The CD44 gene (CDS region sequence) was synthesized according to the human CD44 gene’s mRNA sequence and cloned into the lentiviral vector; this was done for BRG1 as well. Lentiviral vector encoding a short hairpin RNA (shRNA) targeted against BRG1 and a negative control lentiviral vector were also designed and synthesized according to our previous study [20]. Recombinant lentiviruses were produced after the 293 T cells were co-transfected with lentiviral vector and lentiviral packaging plasmids. 293 T cells were obtained from the Cell Resource Center of the Shanghai Institutes for Biological Sciences Type Culture Collection of the Chinese Academy of Sciences (Shanghai, China). Cell lines for stable expression of CD44 or BRG1 were established separately by incubating miR-6511b-5p mimic cells with the infectious lentivirus medium of lenti-CD44 or lenti-BRG1. Besides, Cell transfection was performed using Lipofectamine 2000 reagent (Invitrogen, CA, USA), according to the manufacturer’s protocol.

### Western blot analysis

Western blot analysis was performed according to the protocol of our previous study [20]. Antibodies were purchased as indicated: anti-BRG1 (CAT #49360S), anti-CD44 (CAT #37259S), anti-E-cadherin (CAT #3195S), anti-β-catenin (CAT #8480S), anti-vimentin (CAT #5741S), and anti-N-cadherin (CAT #13116S) (Cell Signaling Technology, MA, USA), and anti-actin (CAT #SAB3500350) (Sigma-Aldrich, MO, USA).

### Transwell invasion assay

To measure cell invasion ability, a total of 4 × 10^5^ cells were suspended in serum-free DMEM and plated in the upper chamber coated with collagen 1 (CAT #C9879, Sigma-Aldrich, MO, USA). A Corning Matrigel invasion chamber with a polycarbonate membrane with an 8 mm pore size was used according to the manufacturer's protocol (Corning‐Costar, MA, USA). First, 0.5 mL DMEM supplemented with 10% FBS was added to the lower chambers. After culturing for 24 h, the assays were stopped and the cells remaining in the upper chamber were removed with cotton swabs. Invaded cells were fixed with 4% paraformaldehyde and stained with crystal violet (CAT #V5265, Sigma-Aldrich, MO, USA) for 20 min. The number of invaded cells in five random fields per chamber was counted using a light microscope at 200 × magnification. All photographs were taken under high magnification using a microscope (Nikon, Tokyo, Japan).

### Wound healing assays

Cells were incubated in 6-well plates for 24 h until a monolayer of cells was formed. Cell monolayers were scratched using a 200 µL pipette tip, and a cell-free uniform gap was created. The plates were then washed twice with PBS and cultured in serum-free DMEM for 24 h. Photos were taken at 0 h and 24 h in the same selected areas. The ratios of wound closure in 24 h were normalized to the gap at 0 h and were calculated after the capture of six random sites.

### Luciferase reporter assay

Dual-luciferase reporter assays (Promega, Madison, WI, USA) were performed in SW620 and HT29 cells, according to the manufacturer’s instructions. The putative binding site of miR-6511b-5p in the 3′-UTR of BRG1 mRNA (WT) or its mutant sequence (MUT) was cloned downstream of the firefly luciferase gene. SW620 and HT29 cells were co-transfected with 50 nM miR-6511b-5p mimics or negative control and 0.5 μg of psiCHECK-2-BRG1-3′-UTR-WT or psiCHECK-2-BRG1-3′-UTR-MUT using Lipofectamine 2000 (Invitrogen, CA, USA). Firefly and Renilla luciferase activities were measured using a GloMax fluorescence reader (Promega, Madison, WI, USA) after 48 h of transfection.

### In situ hybridization (ISH) and immunohistochemistry(IHC)on tissue microarray

Tissue microarrays (TMA) containing 40 colorectal cancer tumor specimens were constructed in cooperation with the Xin Chao Company (Shanghai, China). Tumors were collected from the Department of General Surgery, Shanghai General Hospital, between June 2008 and February 2010. Expression of miR-6511b-5p was detected by in situ hybridization in dMMR and pMMR colorectal cancer tumor tissues. In addition, we assessed BRG1 and CD44 protein expression levels using immunohistochemical staining of TMA. The in situ hybridization and immunohistochemistry assays on TMA were performed according to the protocol described in our previous study [14].

### Chromatin immunoprecipitation assay

In this assay, the ChIP kit (CAT#26156, Thermo Scientific, MA, USA) was used according to the manufacturer’s instructions. In brief, colorectal cancer cells were collected after washed by PBS and fixed by 1% formaldehyde and were homogenized using a bead-beating method. Then ultrasound was performed to broke chromatin DNA to a size of 0.5–1 kb. ChIP assays were performed using the BRG1 antibody (CAT #49360S, Cell Signaling Technology, MA, USA), RNA polymerase II antibody (CAT #ab264350, Abcam, Cambridge, UK), or immunoglobulin G control at 4 °C overnight. Finally, the immunoprecipitated DNA/protein complexes were rinsed and eluted, and then we amplified the eluted DNA before examined by RT-qPCR.

### Statistical analysis

SPSS statistical software program version 22 (SPSS, IL, USA) was used for the statistical analysis. The two-tailed Student’s *t* test was used to determine the statistical significance between the two groups. The *χ*^2^ test or Fisher’s exact test was used to analyze the relationship between miR-6511b-5p and clinicopathological features, including MMR status. The survival rates were calculated using the Kaplan–Meier method, and the differences between the survival curves were examined by log-rank tests. Cox proportional hazards models were applied to estimate the effects of the expression of miR-6511b-5p on survival in univariate and multivariate analyses. Statistical significance was set at *P* < 0.05.

## Results

### miR-6511b-5p was downregulated in pMMR colorectal cancer tissues and cell lines

**Fig. 1:**
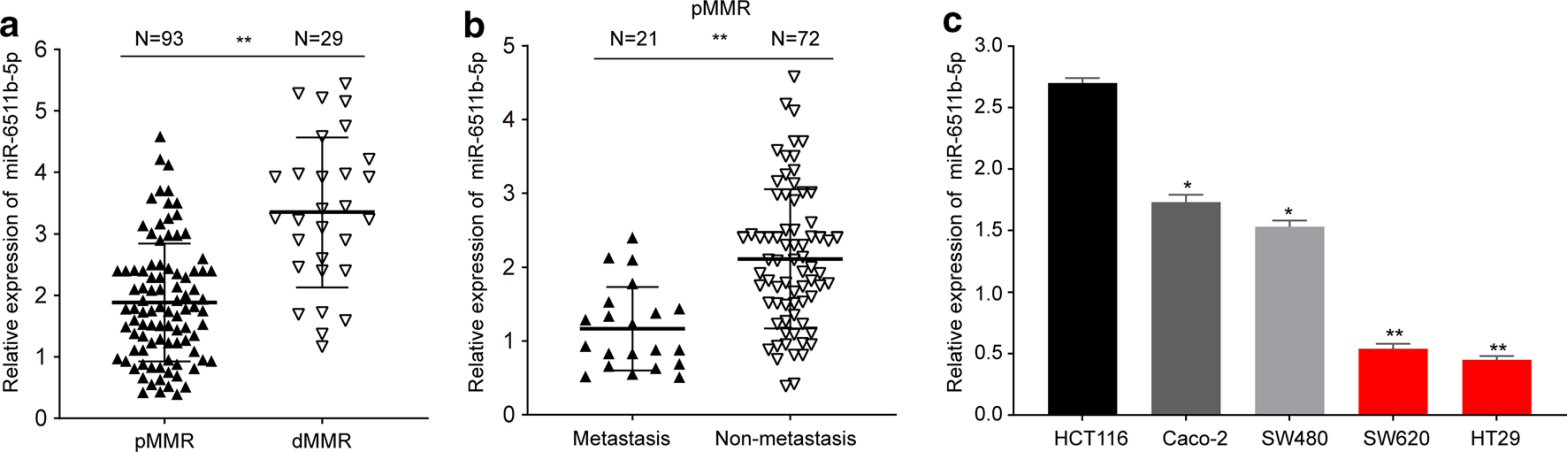
miR-6511b-5p is downregulated in pMMR colorectal cancer tissues and cell lines. **a** The relative expression of miR-6511b-5p was validated in pMMR and dMMR colorectal cancer tissues by qRT-PCR. U6 small nuclear RNA was used as an internal control. **b** miR-6511b-5p expression in metastatic (*n* = 73) and non-metastatic tissues (*n* = 49). **c** The level of miR-6511b-5p in the MSI-H colorectal cancer cell line (HCT116) and four MSS colorectal cancer cell lines (Caco-2, SW480, SW620, and HT29). (*P < 0.05, **P < 0.01)

We validated the expression patterns of 12 miRNAs (miR-1290, miR-664b-5p, miR-3607-5p, miR-3138, miR-298, miR-3653, miR-1291, miR-3679-3p, miR-149-3p, miR-30a-5p, miR-345-3p, miR-6511b-5p) in pMMR colorectal cancer patients by using qRT-PCR. The experiment showed the 12 miRNAs expression profiling of metastatic and non-metastatic pMMR colorectal cancer tissues (Supplementary Fig. S1). It is obvious that miR-6511b-5p appeared to be the most significant identified candidate (*P* < 0.001). To study the role of miR-6511b-5p in colorectal cancer, the expression of miR-6511b-5p was examined by qRT-PCR, and dMMR status was determined by immunohistochemistry (IHC) in 122 patients. As shown in Fig. 1a, miR-6511b-5p expression was lower in pMMR colorectal cancer tissues than in dMMR cancer tissues. Moreover, we also discovered that miR-6511b-5p expression was lower in metastatic pMMR colorectal cancer patients than in non-metastatic cases (Fig. 1b). These results indicated that decreased miR-6511b-5p expression might correlate with metastasis of pMMR colorectal cancer. To further confirm the role of miR-6511b-5p in pMMR colorectal cancer, miR-6511b-5p expression levels were detected in colorectal cancer cells using RT-qPCR. Compared to the MSI-H colorectal cancer cell line HCT116 [23], we found a significant downregulation of miR-6511b-5p in four MSS colorectal cancer cell lines, including SW620, HT29, Caco-2, and SW480, among which miR-6511b-5p levels were the lowest in SW620 and HT29 cells (Fig. 1c). Therefore, we chose SW620 and HT29 cells to study the role of miR-6511b-5p in the invasion and migration of pMMR colorectal cancer cells.

### Decreased expression of miR-6511b-5p was positively correlated with pMMR status and predicted poor prognosis in patients with colorectal cancer

To determine the role of miR-6511b-5p in colorectal cancer, we examined the clinical value of miR-6511b-5p expression by assessing the correlation between miR-6511b-5p expression and the clinicopathological parameters of patients with colorectal cancer. The expression of miR-6511b-5p was examined by qRT-PCR, and the MMR status was determined by IHC in 122 patients with colorectal cancer. As shown in Table 1, the low expression of miR-6511b-5p was positively correlated with pMMR status (*P* = 0.011). In addition, we also found that downregulated miR-6511b-5p expression was closely correlated with T stage (*P* = 0.001), N stage (*P* = 0.007), M stage (*P* < 0.001), AJCC stage (*P* < 0.001), and differentiation (*P* = 0.003) in colorectal cancer patients. These results indicate that downregulation of miR-6511b-5p has potential for the development and metastasis of colorectal cancer.

**Table 1:** Associations between miR-6511b-5p Expression and Clinicopathologic Characteristics (*n* = 122)

Variable	miR-6511b-5p expression		*P* value
	Low (*n* = 75)	High (*n* = 47)	
Age			
< 65 years	32	21	0.827
≥ 65 years	43	26	
Gender			
Male	27	22	0.236
Female	48	25	
T stage			
T1	4	13	0.001*
T2 + T3 + T4	71	34	
N stage			
N0	34	33	0.007*
N1 + N2	41	14	
M stage			
M 0	52	45	< 0.001*
M 1	23	2	
AJCC stage			
I	6	16	< 0.001*
II + III + IV	69	31	
Differentiation			
Well	24	28	0.003*
Moderate + Poorly	51	19	
MMR status			
dMMR	12	17	0.011*
pMMR	63	30	

*P* values are based on Chi-square test or Fisher’s exact test if necessary

*P < 0.05

**Fig. 2:**
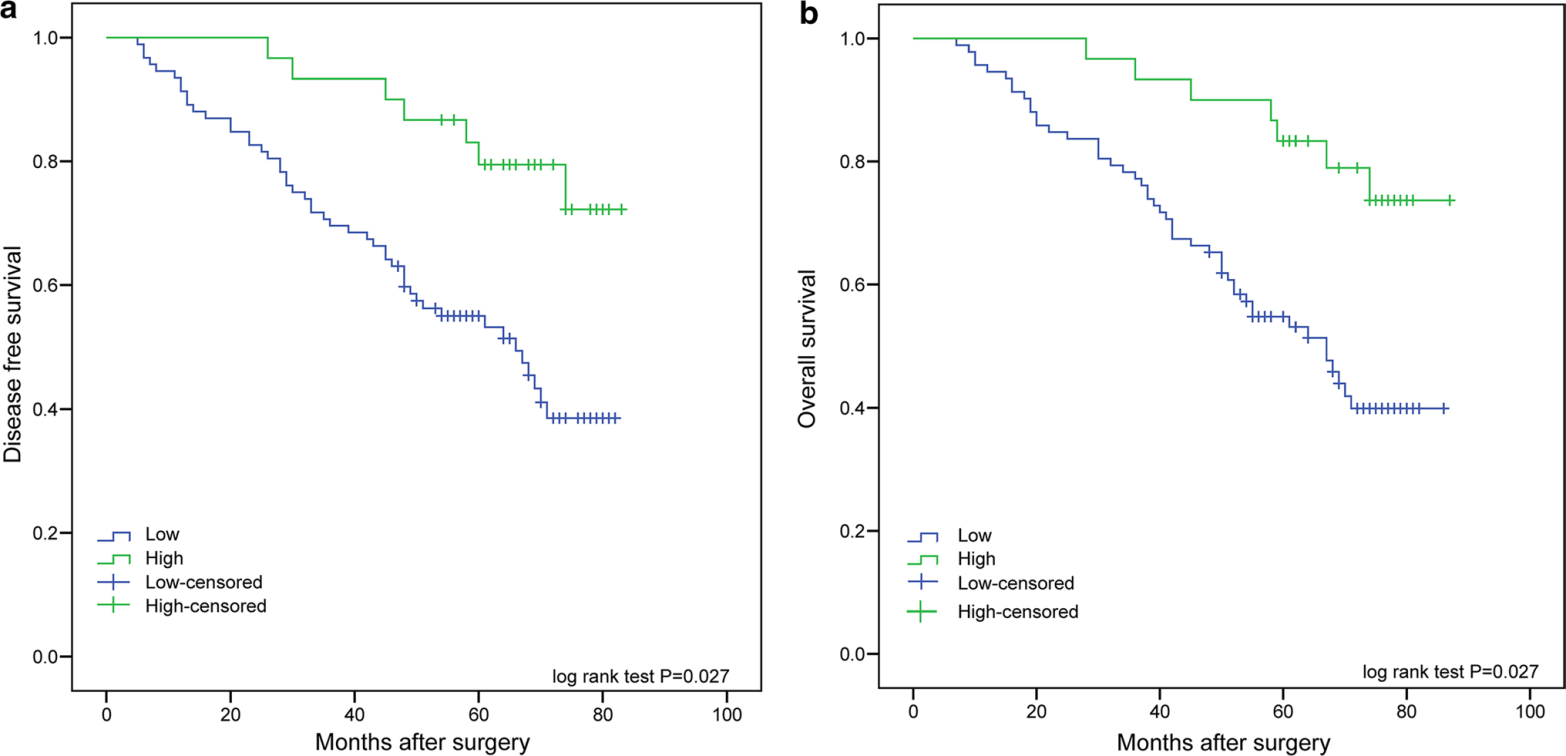
Kaplan–Meier survival analysis of 122 colorectal cancer patients. Overall survival (**a**) and disease-free survival (**b**) were analyzed in patients with different miR-6511b-5 expression

Kaplan–Meier and univariate Cox proportional hazard regression analyses showed that miR-6511b-5p expression was significantly associated with the survival of patients with colorectal cancer. Kaplan–Meier analysis showed that low miR-6511b-5p expression was associated with shorter overall survival (OS) (*P* = 0.027) and disease-free survival (DFS) (*P* = 0.027) than those with higher miR-6511b-5p levels (Fig. 2a and b). Furthermore, univariate and multivariate Cox proportional hazards models were used to examine the predictive value of miR-6511b-5p in patient prognosis after surgery. Using univariate Cox proportional hazard regression analysis, we found that increased lymph node metastasis, distant metastasis, level of tumor differentiation, advanced TNM stage, and decreased miR-6511b-5p expression were all positively associated with reduced OS (Table 2) and DFS (Table 3). We also found that patients with pMMR tumors had a significantly worse OS (HR = 2.534, 95% CI = 1.328–4.835; *P* = 0.005) (Table 2) and DFS (HR = 2.490, 95% CI = 1.306–4.750; *P* = 0.006) (Table 3) compared to patients with dMMR tumors, which is consistent with previous studies [6]. Multivariate analysis was performed using the Cox proportional hazards model for all significant variables in the univariate analysis. However, N stage and distant metastasis were collinear with the AJCC stage, so we excluded them from the final model. The results revealed that miR-6511b-5p expression was an independent prognostic factor for OS (HR = 0.398, 95% CI = 0.166–0.953; *P* = 0.039, Table 2) and DFS (HR = 0.393, 95% CI = 0.165–0.937; *P* = 0.035, Table 3). In addition, MMR status was also shown to be a significant independent prognostic factor for OS (HR = 3.671, 95% CI = 1.835–7.343; *P* < 0.001, Table 2) and DFS (HR = 3.478, 95% CI = 1.750–6.913; *P* < 0.001, Table 3).

**Table 2:** Univariate and multivariate Cox proportional hazard models for overall survival (OS)

Variable	Univariate		Multivariate	
	HR (95% CI)	*P* value	HR (95% CI)	*P* value
Age				
< 65 years	–		NR	
≥ 65 years	1.565 (0.800–3.061)	0.191		
Gender				
Male	–		NR	
Female	1.355 (0.693–2.652)	0.375		
T stage				
T1	–		NR	
T2 + T3 + T4	3.417 (0.822–14.198)	0.091		
N stage				
N0	–		NR	
N1 + N2	8.765 (3.808–20.173)	< 0.001*		
M stage				
M 0	–		NR	
M 1	9.633 (4.981–18.630)	< 0.001*		
AJCC stage				
I	–		–	
II + III + IV	9.967 (1.367–72.667)	0.023*	10.064 (1.284–78.878)	0.028*
Differentiation				
Well	–			
Moderate + Poorly	9.068 (3.206–25.652)	< 0.001*	6.734 (2.344–19.349)	< 0.001*
MMR Status				
dMMR	–		–	
pMMR	2.534 (1.328–4.835)	0.005*	3.671 (1.835–7.343)	< 0.001*
miR-6511b-5p expression				
Low	–		–	
High	0.271 (0.119–0.617)	< 0.001*	0.398 (0.166–0.953)	0.039*

NR variable was not included in the resultant model

*HR* Hazard ratio, *CI* confidence interval

*P < 0.05 indicated that the 95% CI of HR was not including 1

**Table 3:** Univariate and multivariate Cox proportional hazard models for disease-free survival (DFS)

Variable	Univariate		Multivariate	
	HR (95% CI)	*P* value	HR (95% CI)	*P* value
Age				
< 65 years	–		NR	
≥ 65 years	1.610 (0.824–3.148)	0.164		
Gender				
Male	–		NR	
Female	1.324 (0.677–2.589)	0.412		
T stage				
T1	–		NR	
T1 + T3 + T4	3.387 (0.815–14.079)	0.093		
N stage				
N0	–		NR	
N1 + N2	9.579 (4.124–22.247)	< 0.001*		
M stage				
M 0	–		NR	
M 1	11.159 (5.724–21.756)	< 0.001*		
AJCC stage				
I	–		–	
II + III + IV	9.889 (1.356–72.094)	0.024*	9.440 (1.213–73.460)	0.032*
Differentiation				
Well	–		–	
Moderate + poorly	8.547 (3.028–24.128)	< 0.001*	6.174 (2.156–17.676)	0.001*
MMR Status				
dMMR	–		–	
pMMR	2.490 (1.306–4.750)	0.006*	3.478 (1.750–6.913)	< 0.001*
miR-6511b-5p expression				
Low	–		–	
High	0.272 (0.120–0.619)	0.002*	0.393 (0.165–0.937)	0.035*

NR variable was not included in the resultant model

*HR* hazard ratio, *CI* confidence interval

*P < 0.05 indicated that the 95% CI of HR was not including 1

### Restoration of CD44 expression rescued the repressive effect of miR-6511b-5p on metastases in MSS colorectal cancer cell lines

**Fig. 3:**
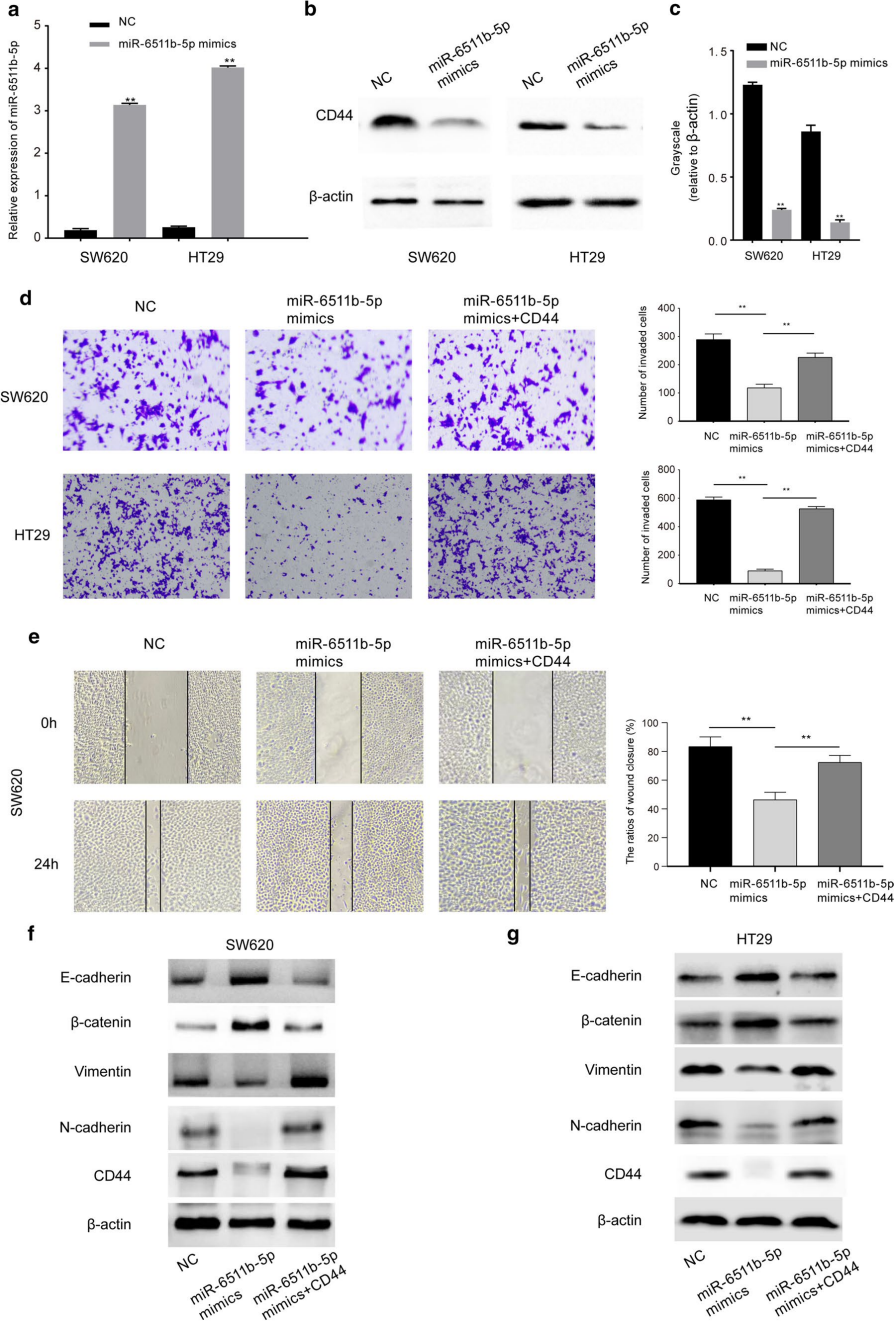
miR-6511b-5p inhibits invasion and migration of MSS colorectal cancer cells via negative regulation of CD44. **a** qRT-PCR analysis of miR-6511b-5p in SW620 and HT29 cells transfected with miR-6511b-5p negative control or miR-6511b-5p mimics. U6 small nuclear RNA was used as an internal control. **b** The expression of CD44 protein in SW620 and HT29 cells with or without miR-6511b-5p overexpression was examined using western blot analysis. **c** Grayscale values were analyzed by Quantity One software (*n* = 3, **P < 0.01). **d** Invasion ability of SW620 and HT29 cells was tested using transwell assays. Histograms show the number of invaded cells. **e** Wound healing assays revealed the impact of miR-6511b-5p and CD44 on the migration ability of SW620 cells. Histograms show the ratio of wound closure. Western blot analysis of EMT-related proteins in SW620 (**f**) and HT29 (**g**) cells with or without miR-6511b-5p overexpression after restoration of CD44 expression. (*p < 0.05, **p < 0.01)

Since distant metastases tend to occur in pMMR colorectal cancer patients with low expression of miR-6511b-5p (Fig. 1b and Table 1), we determined the effect of miR-6511b-5p in MSS colorectal cancer cell metastasis. In addition, miR-6511b-5p expression was deficient in SW620 and HT29 cells, the two cell lines were transfected with miR-6511b-5p mimics to augment their expression, and cells transfected with miR-6511b-5p negative control (NC) were used as controls. As shown in Fig. 3a, miR-6511b-5p mimics strikingly increased the level of endogenous miR-6511b-5p in both cell lines, as expected.

Several previous studies have demonstrated that CD44 enhances colorectal cancer cell migration and invasion and is associated with tumor progression and distant metastasis of colorectal cancer [17, 24]. Thus, we explored whether CD44 is a potential downstream gene of miR-6511b-5p in colorectal cancer metastasis. First, western blot analysis was performed to detect the influence of miR-6511b-5p on the expression of CD44. The results revealed that upregulation of miR-6511b-5p inhibited CD44 expression in both SW620 and HT29 cells (Fig. 3b and c). Subsequently, we explored whether miR-6511b-5p influenced colorectal cancer metastasis in a CD44-dependent manner in SW620 and HT29 cells. As shown in Fig. 3d, the results of the transwell assays showed that overexpression of miR-6511b-5p significantly reduced the invasive abilities of SW620 and HT29 cells. Similarly, compared to miR-6511b-5p NC, overexpression of miR-6511b-5p significantly inhibited the migratory ability of SW620 and HT29 cells (Fig. 3e). In addition, we found that restoration of CD44 expression significantly rescued the repressive impact of miR-6511b-5p on the invasion and migration of SW620 and HT29 cells (Fig. 3d and e). Moreover, western blot analysis was used to detect the expression of proteins associated with epithelial–mesenchymal transition (EMT), a hallmark of metastasis. The reduced expression of epithelial markers, including E-cadherin and β-catenin, as well as the enhanced expression of mesenchymal markers, such as vimentin and N-cadherin, typically appeared in EMT [25, 26]. Similarly, the expression of E-cadherin and β-catenin was remarkably increased, while vimentin and N‐cadherin were clearly decreased in both SW620 and HT29 cells with augmented miR-6511b-5p expression (Fig. 3f and g). We also found that restoration of CD44 expression significantly promoted the EMT process, which was blocked by miR-6511b-5p upregulation in SW620 and HT29 cells (Fig. 3f and g). Overall, our findings demonstrated that miR-6511b-5p inhibited metastasis in MSS colorectal cancer cells via the negative regulation of CD44.

### miR-6511b-5p weakened CD44 expression by directly targeting BRG1

According to many previous studies, miRNAs frequently act as oncogenes or tumor suppressor genes in colorectal cancer progression by inhibiting the transcription of targeted genes. To clarify whether CD44 was a potential target gene of miR-6511b-5p, we performed bioinformatic analyses using miRNA-target-predicting programs (TargetScan, miRabel, and miRWalk). Unfortunately, CD44 did not belong to one of the candidate targets of miR-6511b-5p. Therefore, we speculated whether the expression of CD44 was regulated by target genes of miR-6511b-5p. The SWI/SNF chromatin-remodeling complex plays a crucial role in various cancers and has been demonstrated to be mainly involved in multiple biological processes by remodeling chromatin and regulating transcription [27]. Moreover, the potential binding of miR-6511b-5p with BRG1, the core component of the mammalian SWI/SNF complex, was predicted by miRNA-target-predicting programs (TargetScan, miRabel, and miRWalk). In a previous study, we demonstrated that BRG1 was significantly increased and promoted the invasion and progression of colorectal cancer [20]. As the key regulator of CD44, BRG1 is known to positively regulate the expression of CD44 in several cancer cell lines [18, 28]. However, whether BRG1 is directly targeted by miR-6511b-5p and increases the transcriptional activity of CD44 to promote the invasion and migration of colorectal cancer cells remains unclear.

**Fig. 4:**
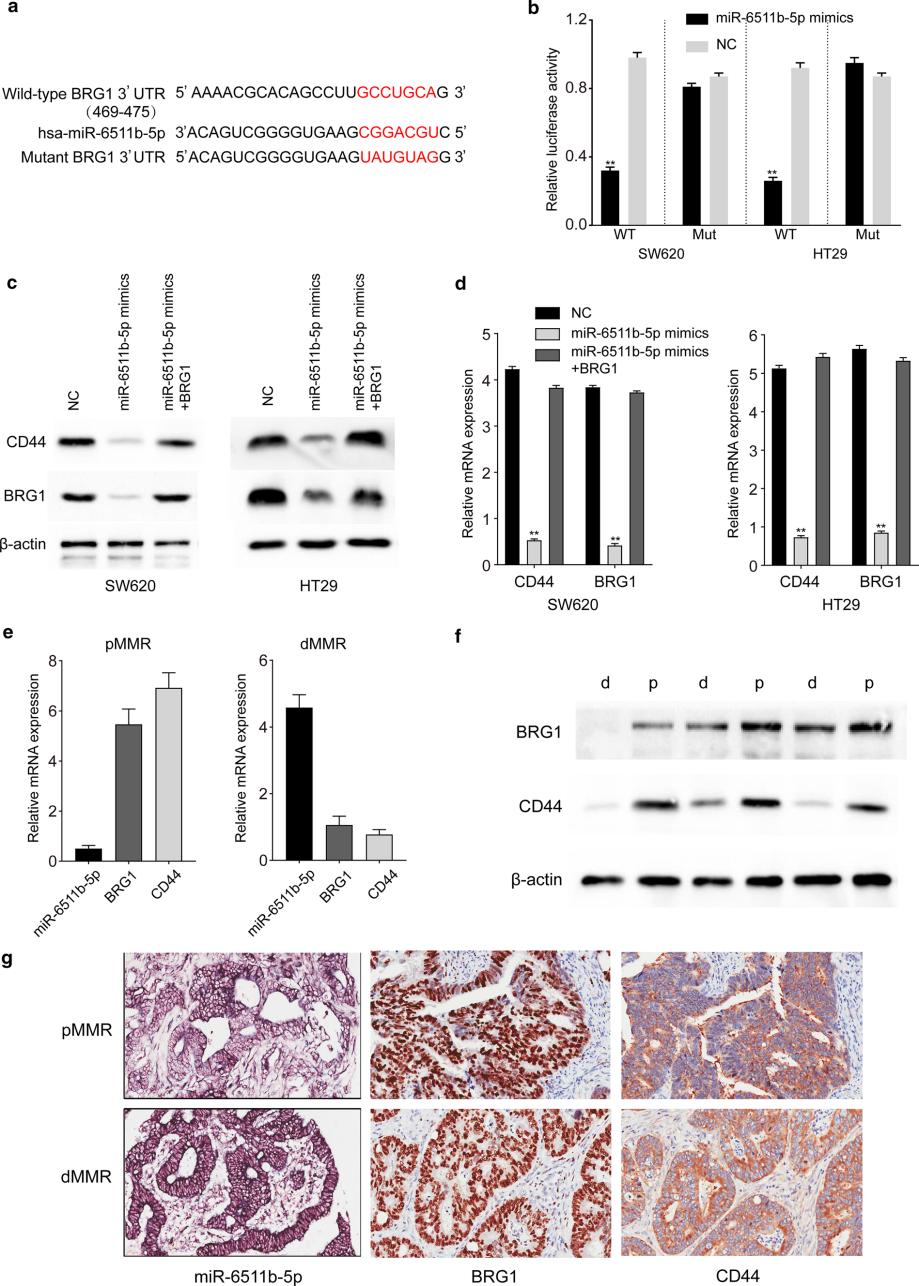
miR-6511b-5p weakened CD44 expression by directly targeting BRG1. **a** RNA sequence alignment showing that the 3′-UTR of BRG1 mRNA contains a complementary site for the seed region of miR-6511b-5p. BRG1 mutant sequence was used as a negative control. **b** Dual-luciferase reporter assay was performed using SW620 and HT29 cells transfected with wild-type or mutated BRG1 reporters plus miR-6511b-5p mimics. The expression of BRG1 and CD44 were respectively identified by western Blot analysis (**c**) and qRT-PCR analysis (**d**) in SW620 and HT29 cells upon different transfections. **e** qRT-PCR analysis of miR-6511b-5p, BRG1 and CD44 in pMMR and dMMR colorectal cancer tissues. **f** Western blot analysis of BRG1 and CD44 proteins in three dMMR and three pMMR representative colorectal tumor tissues. **g** Representative images of miR-6511b-5p, BRG1 and CD44 expression in dMMR and pMMR colorectal cancer tissue microarrays using ISH and IHC staining, respectively. (*P < 0.05, **P < 0.01)

First, to confirm whether BRG1 was directly targeted by miR-6511b-5p, a luciferase reporter containing the complementary seed sequence of miR-6511b-5p at the 3'-UTR of BRG1 mRNA was constructed (Fig. 4a). The luciferase reporter assay indicated that co-transfection with miR-6511b-5p mimics significantly reduced the activity of wild-type BRG1 3'-UTR reporter, but not the mutant BRG1 3′-UTR, both in SW620 and HT29 cells (Fig. 4b). These results indicate that BRG1 is a direct target of miR-6511b-5p. Subsequently, we overexpressed BRG1 in SW620 and HT29 cells, which were transfected with lentiviral vectors containing miR-6511b-5p mimics. Western blot and RT-qPCR analyses revealed that overexpression of BRG1 markedly restored the inhibition of miR-6511b-5p overexpression on BRG1 and CD44 (Fig. 4c and d). Overall, miR-6511b-5p negatively regulated BRG1 and CD44 expression in vitro. In addition, we demonstrated that the expression of BRG1 and CD44 were both upregulated in pMMR colorectal cancer tissues and downregulated in dMMR samples by RT-qPCR and western blot analysis (Fig. 4e and f). In contrast, miR-6511b-5p was downregulated in pMMR colorectal cancer tissues compared to that in dMMR tissues (Fig. 4e). Furthermore, we detected the expression of miR-6511b-5p, BRG1, and CD44 in these samples using in situ hybridization (ISH) and IHC staining, respectively. The results revealed that miR-6511b-5p expression was low, while BRG1 and CD44 expression was high in pMMR colorectal cancer tissues (Fig. 4g). Moreover, the opposite was observed in dMMR tissues. These results suggest that miR-6511b-5p decreases CD44 expression by directly targeting BRG1 in colorectal cancer cell lines and tissues.

### BRG1 enhanced the expression of CD44 by promoting CD44 demethylation in colorectal cancer

**Fig. 5:**
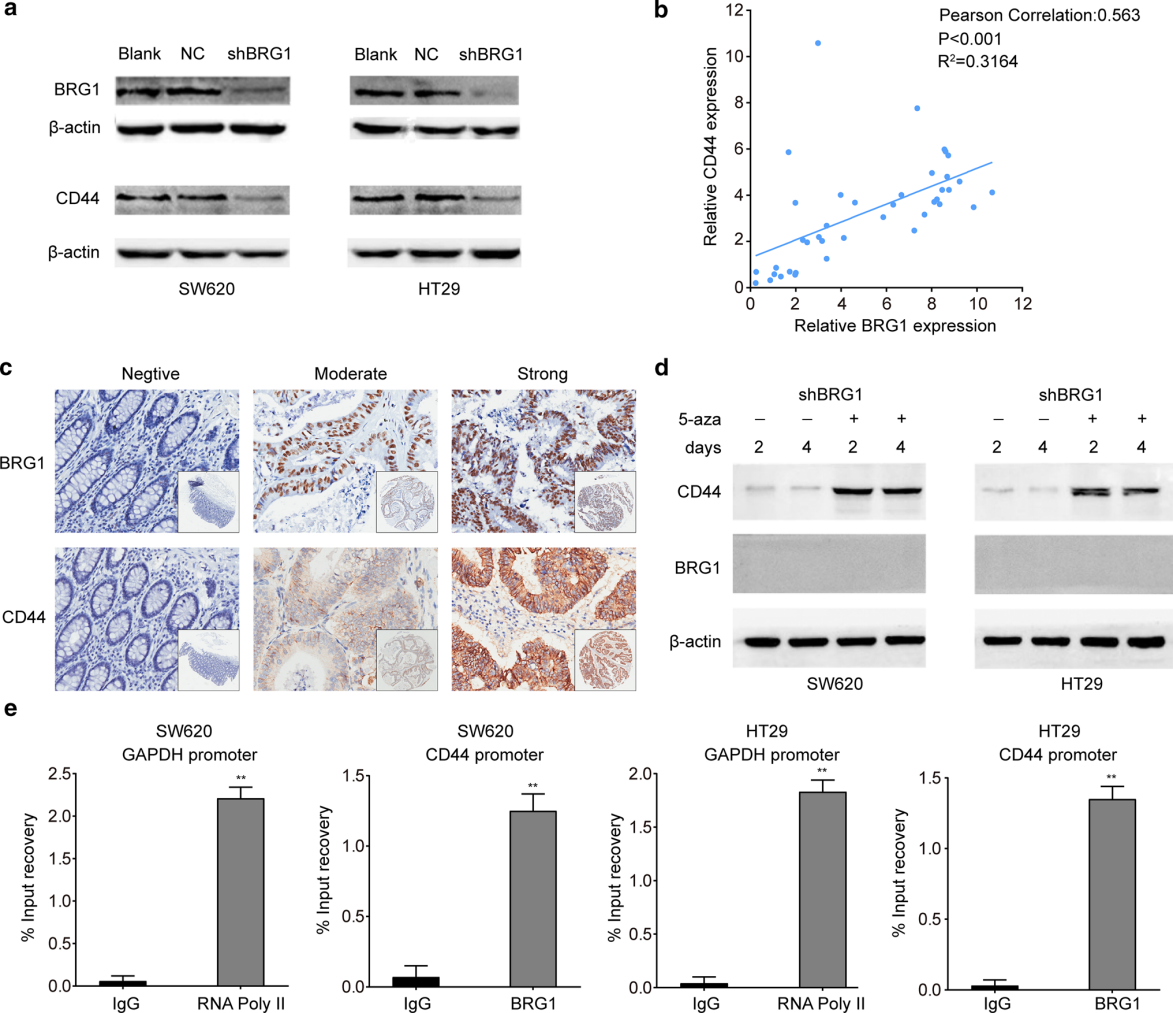
BRG1 enhanced the expression of CD44 by promoting CD44 demethylation in colorectal cancer. **a** Western Blot analysis of BRG1 and CD44 expression in SW620 and HT29 cells after knockdown of BRG1. **b** Positive correlation between BRG1 mRNA levels and CD44 mRNA levels in 40 colorectal cancer tissues (Pearson correlation: 0.563, *P* < 0.001, *R*^2^ = 0.3164). **c** Immunohistochemical staining revealed a positive correlation between BRG1 protein levels and CD44 protein levels in tissue microarrays. **d** Western blot analysis of BRG1 and CD44 proteins in BRG1 knocked down cells which are in the presence of 5-aza for 2 and 4 days. **e** ChIP assays were performed to validate the binding of BRG1 to CD44 promoter in colorectal cancer cells, with the absolute interaction of RNA polymerase II with GAPDH promoter acting as a control. (*P < 0.05, **P < 0.01)

As a component of the SWI/SNF chromatin-remodeling complex, BRG1 is a major regulator of transcription [27]. Previous studies have indicated the necessity of BRG1 for the transcription and expression of CD44 in colorectal cancer [29]. However, the detailed mechanism by which BRG1 modulates CD44 expression in colorectal cancer remains unknown. Western blot analysis revealed that knockdown of BRG1 with shRNA inhibited CD44 expression in both SW620 and HT29 cells (Fig. 5a). Real-time PCR and immunohistochemistry were conducted to assess the expression of BRG1 and CD44 in 40 fresh pMMR colorectal cancer tissues and consecutive tissue microarrays, respectively. The expression of CD44 mRNA was positively correlated with BRG1 in colorectal cancer tissues (Fig. 5b, Pearson correlation: 0.563, *P* < 0.001, *R*^2^ = 0.3164). Immunohistochemical staining also showed similar results; patients whose localized colorectal tumors were BRG1-strong had a significantly higher CD44 expression than those with BRG1-moderate tumors (Fig. 5c). These results were consistent with those of a previous study and suggested that CD44 was most likely regulated by BRG1 in colorectal cancer.

BRG1 influences the methylation of CD44 promoters in several cancer cell lines, such as SW13, C33A, HeLa, and Saos-2, but this phenomenon has not been detected in colorectal cancer cell lines [18]. First, we examined whether the CD44 promoter is hypermethylated in BRG1 knocked down colorectal cancer cell lines. As shown in Fig. 5d, after 2 and 4 days in the presence of 5-aza, which causes genomic hypomethylation by reducing the activity of DNA methyltransferase 1 (DNMT1), CD44 expression was induced in SW620 and HT29 cell lines but not in the absence of 5-aza (Fig. 5d). In contrast, the presence of 5-aza did not affect the expression of BRG1. Thereafter, ChIP assays were performed to validate whether BRG1 interacts directly with the CD44 promoter in colorectal cancer cells. The results showed that the CD44 promoter was harvested in the immunoprecipitations of BRG1 groups in both SW620 and HT29 cell lines, similar to the GAPDH promoter that can be easily captured by RNA polymerase II (Fig. 5e). Collectively, these data indicate that CD44 is hypermethylated in cells that lack BRG1, suggesting that transcriptional activation by BRG1 may be involved in the demethylation of CD44.

## Discussion

MMR is crucial in the development and progression of colorectal cancer, and the detection of MMR status has become increasingly popular in the diagnosis and treatment of colorectal cancer. Compared to dMMR patients, pMMR patients are more likely to develop early metastases and respond poorly to immune checkpoint inhibitors, such as programmed death 1 (PD-1), programmed death-ligand 1 (PD-L1), and cytotoxic T-lymphocyte antigen 4 (CTLA-4) [7, 30]. In addition, the prognosis of metastatic pMMR colorectal cancer patients is extremely poor. To better prevent and treat the metastasis of pMMR colorectal cancer, we explored the underlying mechanisms in the present study.

Although many studies have revealed the expression profiles and mechanisms of miRNAs in colorectal cancer, few studies have focused on the differences between pMMR and dMMR colorectal cancer [31, 32]. In our previous work, differentially expressed miRNAs between four dMMR and four pMMR tumor samples were identified using miRNA microarray analysis [14]. Among these differentially expressed miRNAs, we focused on miR-6511b-5p, which was significantly downregulated in pMMR colorectal cancer cells compared with dMMR cells obtained using laser capture microdissection. Previous studies have demonstrated that miR-6511b-5p was downregulated in serum of colorectal cancer patients [21, 33]. In uterine corpus endometrial carcinoma, higher expression of miR-6511b-5p is associated with an increased risk of death and recurrence by regulating APC2 [34]. In rheumatoid arthritis, miR-6511b-5p in serum exosomes was positively correlated with clinical remission [35]. In addition, miR-6511b-5p was detected up-regulated in nicotine-treated human periodontal ligament cells, but the underlying mechanisms were still unknown [36]. In this study, we explored the potential role of miR-6511b-5p in pMMR colorectal cancer. First, we detected that downregulation of miR-6511b-5p appeared more often in pMMR colorectal cancer patients than in dMMR cases, and was positively correlated with metastases. In addition, lower miR-6511b-5p expression predicted poorer prognosis in colorectal cancer patients. We also demonstrated that pMMR status was correlated with poor prognosis of colorectal cancer, which is consistent with previous studies [6]. Finally, we elucidated the mechanism by which miR-6511b-5p inhibits the migration and invasion of MSS colorectal cancer cells via the BRG1/CD44 axis.

EMT plays an important role in the migration and invasion of epithelial cancer cells [37]. A previous study showed that enhancement of CD44 resulted in EMT changes, including mesenchymal markers and epithelial markers, indicating the possibility that CD44 is involved in EMT in colorectal cancer cells [38]. In this study, we found that upregulation of miR-6511b-5p by miRNA mimics diminished the expression of CD44 and reduced the migratory and invasive ability of colorectal cancer cells. Meanwhile, the upregulation of miR-6511b-5p increased E-cadherin and β-catenin expression and decreased vimentin and N-cadherin expression in colorectal cancer cells. To further prove that CD44 acts as the downstream target of miR-6511b-5p in the process of EMT in colorectal cancer cells, CD44 and miR-6511b-5p were both overexpressed in SW620 and HT29 cells. We found that simultaneous overexpression of CD44 and miR-6511b-5p inhibited E-cadherin and β-catenin expression, but increased vimentin and N-cadherin expression. Collectively, our observations and findings indicate that the metastasis-repressive role of miR-6511b-5p in colorectal cancer is mediated by a CD44-dependent pathway.

It is well known that miRNAs inhibit translation by binding to the 3'UTR of target mRNAs [39]. Although we found that miR-6511b-5p inhibited CD44 expression both in SW620 and HT29 cells, CD44 was not the target gene of miR-6511b-5p, as determined by miRNA-target-predicting programs (TargetScan, miRabel, and miRWalk). As a transcription regulation factor, we demonstrated that BRG1 participates in colorectal cancer metastasis by regulating Wnt3a [20]. BRG1 has also been shown to play a pivotal role in various human cancers by regulating downstream genes [40–42]. In this study, we demonstrated that miR-6511b-5p directly binds to the 3'-UTR of BRG1 and suppresses its expression. Moreover, reintroduction of BRG1 rescued the downregulation of CD44 mediated by miR-6511b-5p. Furthermore, our study also showed that miR-6511b-5p levels were inversely correlated with both BRG1 and CD44 expression in colorectal cancer tumor samples. In addition, we confirmed that downregulated miR-6511b-5p was associated with pMMR status and upregulated miR-6511b-5p was associated with dMMR status, as determined by in situ hybridization. Taken together, these results demonstrate that miR-6511b-5p inhibits the expression of CD44 by directly targeting BRG1 in colorectal cancer.

Previous studies have shown that BRG1 is critical for the expression of CD44 in several cancer cells and demonstrated that CD44 expression is induced by the demethylating agent 5-aza in BRG1 deficient cancer cells [18, 19]. Nonetheless, whether BRG1 promotes the expression of CD44 by demethylating the promoter of the CD44 gene in colorectal cancer requires further study. In this study, we analyzed the protein and mRNA expression of BRG1 and CD44 and showed a positive correlation between BRG1 and CD44 expression in both colorectal cancer tissues and cell lines. Moreover, we demonstrated that the loss of BRG-1 correlates with a lack of CD44 expression by influencing the methylation of the CD44 promoter in colorectal cancer cell lines, which could be restored in the presence of 5-aza.

The underlying metastasis-related mechanisms of MMR status in colorectal cancer remain unclear. Our study shows that downregulated miR-6511b-5p is a promising biomarker for the diagnosis of pMMR colorectal cancer, especially in metastatic cases, and predicts poor prognosis in colorectal cancer patients. Although we examined miR-6511b-5p expression in 122 patients, this sample size was not sufficient to support the clinical use of miR-6511b-5p. Thus, a larger clinical sample size study is required to validate our results regarding miR-6511b-5p expression in patients with colorectal cancer. In addition, this is an in vitro mechanism study, which still lacks in vivo validation, and we will improve the pertinent research content in subsequent studies.

In general, we found that miR-6511b-5p suppresses colorectal cancer cells invasion and migration through impairing CD44 expression via directly targeting BRG1. Further research suggested that BRG1 demethylates the promoter of CD44 and influences its transcription and expression. This study identified that miR-6511b-5p may act as a promising biomarker and treatment target for pMMR colorectal cancer, particularly in metastatic patients.

## Supplementary Information

Below is the link to the electronic supplementary material.

Supplementary Figure S1. Validation of the expression patterns of 12 miRNAs in pMMR colorectal cancer tissues using qRT-PCR. The expression of miR-6511b-5p, miR-1290, miR-3138, miR-298, miR-3653, miR-1291, miR-149-3p and miR-30a-5p were significantly lower in metastatic pMMR colorectal cancer tissues compared with non-metastatic cases. (p < 0.05). MiR-6511b-5p appeared to be the most significant identified candidate. (p<0.001). U6 small nuclear RNA was used as an internal control. (TIF 6443 KB)

Supplementary file2 (TXT 392 KB)
