# Supplementary figures and images for: MiR-6511b-5p suppresses metastasis of pMMR colorectal cancer through methylation of CD44 by directly targeting BRG1
Source: Clin Transl Oncol. 2022 May 19;24(10):1940–53. doi: 10.1007/s12094-022-02845-4 (PMC9418090; doi:10.1007/s12094-022-02845-4)

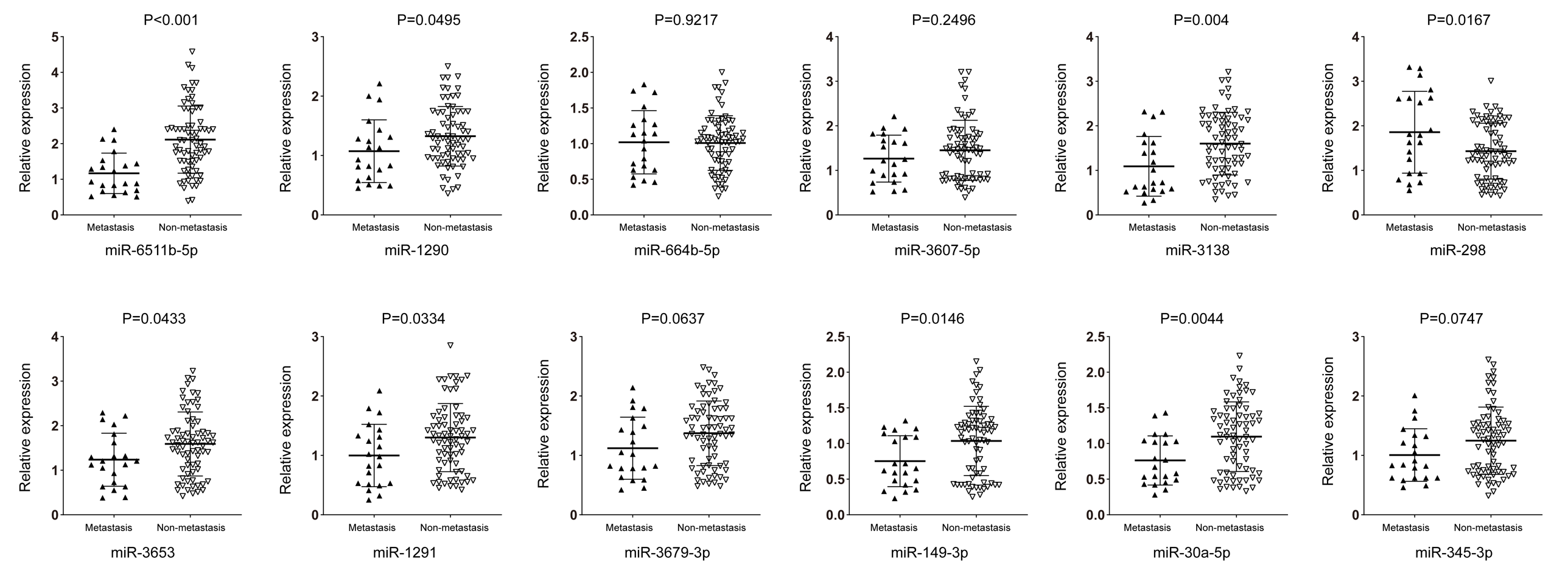

Supplement: Supplementary file 1 — Supplementary Figure S1. Validation of the expression patterns of 12 miRNAs in pMMR colorectal cancer tissues using qRT-PCR. The expression of miR-6511b-5p, miR-1290, miR-3138, miR-298, miR-3653, miR-1291, miR-149-3p and miR-30a-5p were significantly lower in metastatic pMMR colorectal cancer tissues compared with non-metastatic cases. (p < 0.05). MiR-6511b-5p appeared to be the most significant identified candidate. (p<0.001). U6 small nuclear RNA was used as an internal control. (TIF 6443 KB) [file 12094_2022_2845_MOESM1_ESM.tif]
